# Self-doped molecular Mott insulator for bilayer high-temperature superconducting La_3_Ni_2_O_7_

**DOI:** 10.1093/nsr/nwaf353

**Published:** 2025-08-22

**Authors:** Zhan Wang, Heng-Jia Zhang, Kun Jiang, Fu-Chun Zhang

**Affiliations:** Beijing National Laboratory for Condensed Matter Physics and Institute of Physics, Chinese Academy of Sciences, Beijing 100190, China; Kavli Institute for Theoretical Sciences, University of Chinese Academy of Sciences, Beijing 100190, China; Kavli Institute for Theoretical Sciences, University of Chinese Academy of Sciences, Beijing 100190, China; Beijing National Laboratory for Condensed Matter Physics and Institute of Physics, Chinese Academy of Sciences, Beijing 100190, China; School of Physical Sciences, University of Chinese Academy of Sciences, Beijing 100190, China; Kavli Institute for Theoretical Sciences, University of Chinese Academy of Sciences, Beijing 100190, China

**Keywords:** molecular Mott insulator, high temperature superconductivity, nickelates

## Abstract

The bilayer structure of the recently discovered high-temperature superconducting nickelate La$_3$Ni$_2$O$_7$ provides a new platform for investigating correlation and superconductivity. Starting from a bilayer Hubbard model, we show that there is a molecular Mott insulator limit formed by the bonding band owing to Hubbard interaction *U* and large inter-layer coupling. This molecular Mott insulator becomes self-doped due to electrons transferred to the anti-bonding bands at a weaker inter-layer coupling strength. The self-doped molecular Mott insulator is similar to the doped Mott insulator studied in cuprates. We propose La$_3$Ni$_2$O$_7$ to be a self-doped molecular Mott insulator, whose molecular Mott limit is formed by two nearly degenerate anti-symmetric $d_{x^2-y^2}$ and $d_{z^2}$ orbitals. Partial occupation of the higher-energy symmetric $d_{x^2-y^2}$ orbital leads to self-doping, which may be responsible for high-temperature superconductivity in La$_3$Ni$_2$O$_7$. The effects of Hund’s coupling $J_H$ on the low-energy spectra are also studied via exact diagonalization. The proposed low-energy theory for La$_3$Ni$_2$O$_7$ is found to be valid for a wide range of *U* and $J_H$.

## Teaser

In the bilayer high-temperature superconducting La3Ni2O7 system, a model of self-doped molecular Mott insulator, where the Mott limit is given by two nearly degenerate interlayer antisymmetric dx2-y2 and dz2 molecular orbitals with strong repulsion accompanied by the self-doping effect owing to the symmetric orbital, is proposed to describe the low-energy physics and accounting for the mechanism of its high-temperature superconductivity.

## INTRODUCTION

The discovery of high-temperature superconductivity in cuprates greatly challenges our understanding of condensed matter physics [1]. The underlying physics of cuprates is widely believed to be deeply related to electron correlation and their parent Mott insulator states [2,3]. Hence, doping a Mott insulator is one central role for realizing a high-temperature superconductor. Following this idea, it was proposed that doping the Mott nickelates could also lead to high-temperature superconductivity [4]. This idea became realized in thin films of ‘infinite-layer’ nickelates (Sr,Nd)NiO$_2$ in 2019 [5–7], which opened the nickel age of superconductivity [8]. Recently, a new type of nickelate, La$_3$Ni$_2$O$_7$ (LNO), was successfully synthesized with a high-temperature superconducting transition $T_c\sim 80$ K under high pressure [9–14]. Many theoretical proposals have been developed to uncover its superconducting mechanism [15–31].

Compared to the essential CuO$_2$ layer in cuprates, the central ingredient for La$_3$Ni$_2$O$_7$ is the bilayer NiO structure. The valence charge of Ni is 3d$^{7.5+}$, seemingly far away from any strong correlation limit. It is then interesting and important to establish the Mott limit of the bilayer system. Bilayer systems were theoretically explored many years ago, especially the bilayer Hubbard model [32–39]. In this work, we wish to show that the strong correlation limit of this bilayer structure is described by the two-orbital molecular Mott insulator, and that self-doping this molecular Mott insulator leads to high-temperature superconductivity.

The content of this paper is organized as follows. Firstly, we demonstrate that in the bilayer system with inter-layer hopping, the corresponding Mott limit can be addressed under the basis of inter-layer molecular orbitals. In addition, a self-doping regime is expected when the molecular gap is small. Next, in the case of La$_3$Ni$_2$O$_7$, we show that the local electronic structure is described by the two sets of $e_g$ orbitals from each layer. The two $e_g$ orbitals maintain distinct inter-layer hopping amplitudes, resulting in the two-orbital molecular Mott insulator of the anti-symmetric $3d_{z^2}$ and $3d_{x^2-y^2}$ molecular orbitals with total filling equal to one. Furthermore, self-doping to the two-orbital molecular Mott insulator is introduced due to electrons occupying the symmetric $3d_{x^2-y^2}$ orbital at higher energy. The proposed scenario is further demonstrated by solving the local electronic states using exact diagonalization at realistic parameters. The superconducting pairing symmetry is also analyzed using renormalized mean field theory.

## RESULTS

### Self-doped molecular Mott insulator

**Figure 1.**
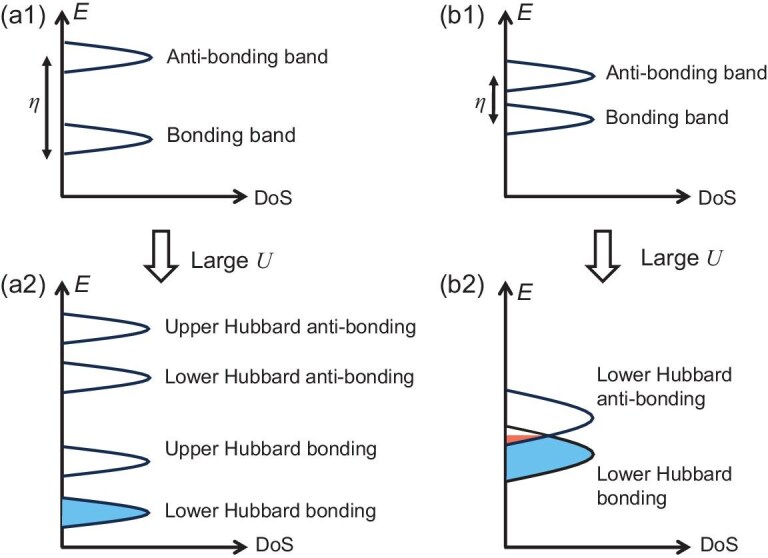
Self-doped molecular Mott insulator. Panels (a1) and (b1) show bands formed by molecular bonding and anti-bonding orbitals that are separated by different values of the molecular orbital splitting $\eta$. Panels (a2) and (b2) schematically show the upper and lower Hubbard bands resulting from onsite repulsion *U* in different limits of $\eta /U$. The filling considered here is one electron per molecule. For $\eta \gg U$ as shown in (a2), the four Hubbard bands are isolated from each other and the electrons will fill the lower Hubbard bonding band only. This corresponds to the scenario of a molecular Mott insulator. In the scenario of (b2) where $\eta < U$, overlap between the lower Hubbard bands of the two molecular orbitals is found due to small $\eta$. The two upper Hubbard bands at higher energy are empty and are not plotted here. At the quarter filling, the electrons now reside primarily in the lower Hubbard bonding band, with a small portion in the lower Hubbard anti-bonding band, giving rise to the self-doped molecular Mott insulator.

To introduce the concept of the self-doped molecular Mott insulator, we start by considering two identical layers of single-band electrons stacked together to form a bilayer system with inter-layer hopping parameter, denoted ${\eta }/2$. In the non-interacting limit, the inter-layer hopping separates the electron dispersion into a bonding and an anti-bonding band, as illustrated in Fig. 1a1 and b1. The Wannier orbitals for the bonding and anti-bonding bands are linear combinations of atomic orbitals belonging to different layers. Hence, these Wannier orbitals should be considered as *molecular orbitals*.

Next, taking the Hubbard interaction *U* into account, the electron spectrum splits into the upper and lower Hubbard bands. The inter-layer hopping ${\eta }/2$ competes with the onsite repulsion *U* and the bandwidth, leading to different insulating limits beyond the upper and lower Hubbard bands in the single-band Hubbard model. There are two typical scenarios, as schematically illustrated in Fig. 1. In the first case where $\eta$ is large, as depicted in Fig. 1a2, the four Hubbard bands are well separated from each other. At the quarter filling of one electron per molecule, we get the singly occupied lower Hubbard bonding band. The effective theory, in terms of molecular orbitals, shares the same feature as the single-band Mott insulator. Therefore, it is a *molecular Mott insulator*. An intriguing scenario is expected when the molecular energy splitting $\eta$ is not too large, as shown in Fig. 1b2. In this case, the two lower Hubbard bands of the bonding and anti-bonding orbitals overlap. Consequently, a small number of electrons can occupy the Hubbard anti-bonding band. Consequently, the Hubbard bonding band becomes hole doped even at the quarter filling. We term this scenario the *self-doped molecular Mott insulator*.

**Figure 2.**
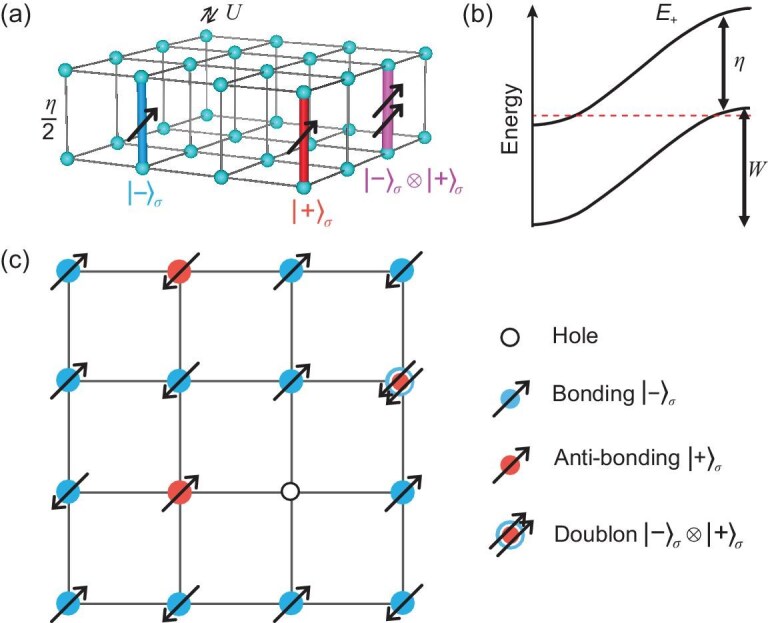
Self-doped molecular Mott insulator from the bilayer Hubbard model. (a) Schematic illustration of the bilayer Hubbard model with inter-layer hopping, denoted by ${\eta }/{2}$, intra-layer hopping *t* and onsite repulsion *U*. The bonding and anti-bonding orbitals in the molecular basis are shown in blue and red, respectively. The doublon state is shown in purple. (b) Schematic energy dispersion of the bonding and anti-bonding bands. At $\eta <W$, the ground state at the filling of one electron per molecule contains both the bonding and anti-bonding orbitals. (c) Schematic illustration of the self-doped molecular Mott insulator with large *U*. The blue and red circles denote the electrons in the bonding and anti-bonding orbitals, respectively. Note that the depicted doublon state with both orbitals occupied does not cost *U* and is also allowed in the low-energy range; see equation (4).

As a concrete model to realize the self-doped molecular Mott insulator, consider the bilayer square lattice Hubbard model depicted in Fig. 2a. The Hamiltonian can be written as


(1) \begin{eqnarray*}
H_{\text{bilayer}}=H_t+H_U
\end{eqnarray*}

with the kinetic part $H_t$ and the interaction part $H_U$ given by


(2) \begin{eqnarray*}
H_t = -t\sum _{\langle ij\rangle \sigma }\sum _{l=t,b}c_{il\sigma }^\dagger c_{jl\sigma }+\frac{\eta }{2}\sum _{i,\sigma } c_{it\sigma }^\dagger c_{ib\sigma }+\text{H.c.},\\
\end{eqnarray*}

(3) \begin{eqnarray*}
H_U = U\sum _{i}\sum _{l=t,b} n_{il\uparrow }n_{il\downarrow }.
\end{eqnarray*}

Here the layer-*l* indices *t* and *b* denote the top and bottom layers, respectively; $c_{il\sigma }^\dagger$ creates an electron with spin $\sigma$ at planar site *i* of layer *l* and $n_{il\sigma }$ is the corresponding electron number operator. The two layers share the same in-plane nearest-neighbor hopping amplitude *t* and repulsive Hubbard interaction *U*. The inter-layer hopping is ${\eta }/{2}$ between the corresponding sites from the two layers. We assume that $\eta >0$.

The kinetic part $H_t$ can be diagonalized under the basis of the inter-layer molecular bonding (anti-symmetric, ‘$-$’) and anti-bonding (symmetric, ‘$+$’) orbitals, namely, $c_{i\pm \sigma }=(c_{it\sigma }\pm c_{ib\sigma })/\sqrt{2}$, as shown in blue and red in Fig. 2a. The obtained molecular energy dispersions are $E_{k\pm }=\epsilon _k\pm {\eta }/{2}$ with $\epsilon _k=-2t[\cos k_x+\cos k_y]$. The molecular energy splitting between the bonding and anti-bonding bands is $\eta$. The bonding band ${E_{k-}}$ has lower energy, as schematically depicted in Fig. 2b. The band width *W* is determined by the in-plane hopping and the band energy splitting corresponds to $\eta$. For $\eta \lesssim W$, there is a finite overlap between the bonding and anti-bonding bands, as highlighted by the red dashed line in Fig. 2b.

At large Hubbard *U*, the electron band further splits into the upper and lower Hubbard bands. If $\eta \lesssim W\ll U$, we expect that the local electrons are mostly found in the lower Hubbard bonding band $E_-$, with a small number of electrons in the Hubbard anti-bonding band $E_+$, realizing the self-doped molecular Mott insulator through the scenario similar to that depicted in Fig. 1b.

It is well known that the doped Mott insulator can be well described by the effective *t*-*J* model [2,3,40]. For this self-doped molecular Mott insulator, we can also write down an effective *t*-*J* model. Compared to the single-band *t*-*J* model, there are four possible energy-allowed states: hole, singly occupied anti-bonding $|+\rangle _\sigma$, singly occupied bonding $|-\rangle _\sigma$ and a special doublon state $|+\rangle _\sigma \otimes |-\rangle _\sigma$, as summarized in Fig. 2c. Note that this doublon state is formed by two electrons of the same spin occupying both the bonding and anti-bonding orbitals. This particular configuration is found to be immune from double onsite occupancy constraints as it does not cost *U*. One can see this through its wave function:


(4) \begin{eqnarray*}
c_{i+\sigma }^\dagger \otimes c_{i-\sigma }^\dagger &=& \frac{1}{\sqrt{2}} \left(c_{it\sigma }^\dagger +c_{ib\sigma }^\dagger \right)\otimes \frac{1}{\sqrt{2}} \left(c_{it\sigma }^\dagger -c_{ib\sigma }^\dagger \right)\\
&=& c_{it\sigma }^\dagger \otimes c_{ib\sigma }^\dagger .
\end{eqnarray*}

In this state, one electron is on the top and the other electron is on the bottom layer, which does not cost any *U*. At quarter filling, the total electron number equals one per molecule. The formation of a doublon will generate a hole at another molecular site, as shown in Fig. 2c, leading to the picture of the self-doping effect in real space.

Therefore, the effective *t*-*J* model can be written as, in addition to a weakly correlated band for $|+\rangle _\sigma$ electrons,


(5) \begin{eqnarray*}
H_{t-J} &=& -t^- \sum _{\langle ij\rangle ,\sigma } \hat{P}_G \left(c_{i-\sigma }^\dagger c_{j-\sigma }+\text{H.c.}\right)\hat{P}_G\\
&& +\,J \sum _{\langle ij\rangle }\boldsymbol {S}_{i-}\cdot \boldsymbol {S}_{j-}.
\end{eqnarray*}

Here $t^-$ is the bare hopping amplitude associated with the anti-symmetric bonding electrons; $\boldsymbol {S}_{i-}=\frac{1}{2}c_{i-s_1}^\dagger \boldsymbol {\sigma }_{s_1s_2} c_{i-s_2}$ denotes the local spin operator of the bonding electron,where $s_1,s_2$ represent the spin-$1/2$ indices; $\hat{P}_G$ is the projection operator to the above energy-allowed configurations in the large-*U* limit. Because the correlation effect in anti-bonding orbitals is weak, we only treat them as self-doping to the bonding *t*-*J* model. Owing to the self-doping effect, the *t*-*J* model can naturally induce a *d*-wave superconductivity, as widely studied in cuprates [2,3,41].

### Effective model for $\text{La}_3\text{Ni}_2\text{O}_7$

Next, we turn to the effective model for La$_3$Ni$_2$O$_7$. As discussed in the INTRODUCTION, the La$_3$Ni$_2$O$_7$ material becomes superconducting at high pressure. In comparison to the low-pressure density wave phase, the key to superconductivity is stabilizing its high-pressure structure. The crystal structure of the La$_3$Ni$_2$O$_7$ material under high pressure has space group $Fmmm$, with stacked bilayer NiO$_2$ planes formed by corner-sharing Ni-O octahedra along the apical direction. The Ni–O–Ni angle between two adjacent bilayer octahedra is 180$^{\circ }$ instead of 168$^{\circ }$ at low pressure [9]. This point of view has been further confirmed in recent superconducting thin-film La$_3$Ni$_2$O$_7$ [42–45]. In this work, we focus on the superconducting phase and leave the low-pressure and high-pressure competition to future work.

**Figure 3.**
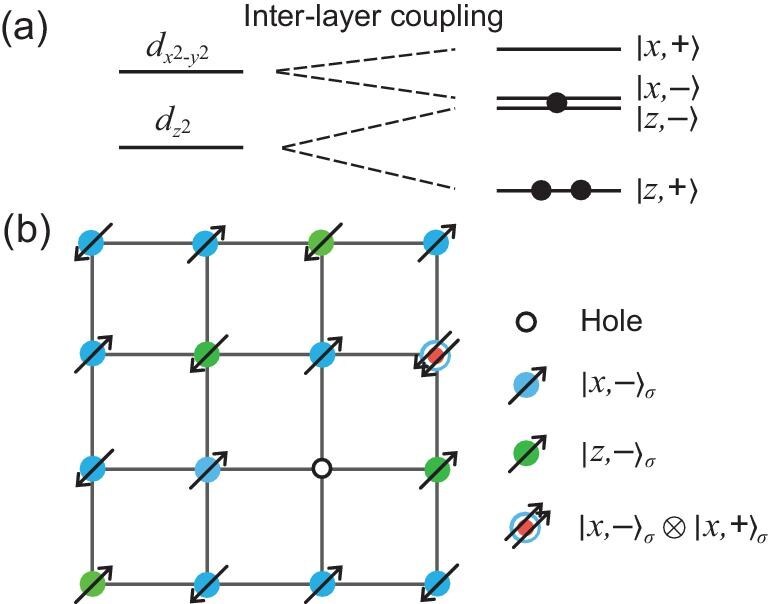
Model for La$_3$Ni$_2$O$_7$: two-orbital self-doped molecular Mott insulator. (a) Local electronic orbitals of the inter-layer pair $(\text{Ni}_2)^{5+}$. For each Ni atom, $3d$  $t_{2g}$ is fully filled and not plotted here. With inter-layer coupling $t_\perp ^{x,z}$, the two sets of $e_g$ orbitals further split into four molecular orbitals. Two electrons occupy the $|z,+\rangle$ orbital that is much lower in energy than the other three orbitals. The remaining one electron predominantly occupies one of the two anti-bonding orbitals $|x,-\rangle _\sigma$ and $|z,-\rangle _\sigma$, with a small portion in the bonding orbital $|x,+\rangle _\sigma$, corresponding to the $\alpha$ band in the DFT calculation [15]. (b) Schematic illustration of the self-doped molecular Mott insulator. In the Mott limit, electrons can only occupy either one of the $|x,-\rangle _\sigma$ and $|z,-\rangle _\sigma$ orbitals due to the strong onsite Hubbard repulsion, as depicted in blue and green, respectively. A small amount of doublon states, namely, $|x,-\rangle _\sigma \otimes |x,+\rangle _\sigma$ as depicted with blue-red circles, can exist due to no cost in *U*. With the total number of electrons fixed, formation of a doublon will generate a hole, leading to the self-doped molecular Mott insulator.

Because of the octahedra crystal field and Jahn–Teller distortion, the partially filled $e_g$ orbitals form the atomic occupancy of bilayer Ni$^{2.5+}$ atoms, as illustrated in Fig. 3a. Owing to the different wave-function symmetries of the $d_{z^2}$ and $d_{x^2-y^2}$ orbitals along the *z* direction, the inter-layer hoppings $t_\perp ^{x,z}$ are different. For $d_{z^2}$ orbitals, the inter-layer hopping is mediated by the $p_z$ orbital of the apical oxygen ions between the top (*t*) and bottom (*b*) layers. The relevant hopping path is $d_{z^2,t}\rightarrow p_z\rightarrow d_{z^2,b}$. The wave-function symmetry of the O-$p_z$ orbital yields a sign change, resulting in $t^z_\perp <0$. For $d_{x^2-y^2}$ orbitals, the same hopping path $d_{x^2-y^2,t}\rightarrow p_z\rightarrow d_{x^2-y^2,b}$ gives zero amplitude and the inter-layer hopping is mediated by other paths including more sites. One can therefore expect the inter-layer hopping $|t_{\perp }^x|<|t_{\perp }^z|$. Consequently, the molecular orbitals formed by the inter-layer (anti-)symmetric combinations of the atomic orbitals maintain different molecular gaps, as depicted in Fig. 3a. More concrete values of the inter-layer hoppings require detailed calculations and we take $t_\perp ^x>0$, as obtained from the density functional theory (DFT) results [46].

In La$_3$Ni$_2$O$_7$, the low-energy physics is described by three electrons per Ni$_2$ molecular ion, filling the two sets of $e_g$ orbitals. The molecular bonding orbital of the $d_{z^2}$ is a symmetric combination of the inter-layer states, denoted $|z,+\rangle$, which has much lower energy and is fully occupied. We therefore drop this orbital in the following discussions in this section. It is interesting that the energy splitting due to Jahn–Teller distortions is comparable to the $t_{\perp }^{z}$ in La$_3$Ni$_2$O$_7$, as reported in [47]. This leads to the nearly degenerate anti-symmetric $e_g$ orbitals, denoted $|x,-\rangle$ and $|z,-\rangle$. It is well known that the two $e_g$ orbitals have a Mott limit at quarter filling [48,49]. The two anti-symmetric molecular orbitals hence form a molecular Mott insulator and represent a two-orbital version of Fig. 1. Furthermore, the symmetric $|x,+\rangle$ orbital is slightly higher in energy than $|x,-\rangle$, introducing self-doped holes into the two-orbital molecular Mott insulator. We show that self-doping this molecular Mott insulator owing to $|x,+\rangle$ gives us the physics of La$_3$Ni$_2$O$_7$.

The Hamiltonian of La$_3$Ni$_2$O$_7$ contains three parts: $H=H_S+H_A+H_{SA}$. For the symmetric orbital $|x,+\rangle _\sigma$, Hamiltonian $H_S$ reads


(6) \begin{eqnarray*}
H_S &=& -\sum _{\langle ij\rangle ,\sigma }t_{ij}^+ \left(c_{i,x+,\sigma }^\dagger c_{j,x+,\sigma }+\text{H.c.}\right)\\
&&+\, \epsilon _{x+}\sum _{i\sigma }c_{i,x+,\sigma }^\dagger c_{i,x+,\sigma },
\end{eqnarray*}

where $c_{i,x+,\sigma }^\dagger$ is the creation operator of $|x,+\rangle _\sigma$ with spin $\sigma$, $t_{ij}^+$ denotes the in-plane hopping amplitude and $\epsilon _{x+}>0$ denotes the molecular energy shift due to $t_\perp ^x$.

Hamiltonian $H_A$ is for anti-symmetric orbitals $|x,-\rangle _\sigma$ and $|z,-\rangle _\sigma$, which we assume to be degenerate for simplicity:


(7) \begin{eqnarray*}
H_A &=&-\sum _{\langle ij\rangle ,\alpha \alpha ^\prime ,\sigma }t_{ij}^{\alpha \alpha ^\prime } \left(c_{i,\alpha -,\sigma }^\dagger c_{j,\alpha ^\prime -,\sigma }+\text{H.c.}\right)\\
&&+\, \sum _{i\alpha }U_A^{\alpha \alpha } n_{i,\alpha -,\uparrow } n_{i,\alpha -,\downarrow } \\
&&+ \sum _{i} U_A^{xz} (n_{i,x-,\uparrow }+n_{i,x-,\downarrow })(n_{i,z-,\uparrow }+n_{i,z-,\downarrow }).\\
\end{eqnarray*}

Here $c_{i,\alpha -,\sigma }^\dagger$ with $\alpha =x,z$ is the creation operator associated with $|x,-\rangle _\sigma$ and $|z,-\rangle _\sigma$ and $n_{i,\alpha -,\sigma }$ is the electron number operator. The in-plane hopping amplitudes $t_{ij}^{\alpha \alpha ^\prime }$ correspond to the electrons hopping between the nearest-neighbor anti-symmetric molecular orbitals $|\alpha ,-\rangle$ and $|\alpha ^\prime ,-\rangle$. The Hubbard interactions between electrons in the anti-symmetric molecular orbitals $|\alpha ,-\rangle$ and $|\alpha ^\prime ,-\rangle$ are denoted by $U_A^{\alpha \alpha ^\prime }$, which are related to the onsite repulsion *U* for the atomic orbitals. We ignore Hund’s coupling term and will come back to its effects in the next section. Note that $H_{SA}$ denotes the coupling between the symmetric and anti-symmetric orbitals, in which the pair hopping term vanishes due to the symmetry.

In the large-*U* limit, Hamiltonian $H_A$ alone describes a molecular Mott insulator with two nearly degenerate orbitals at one-electron filling. This is further justified through solving the local electronic structure of the two sets of $e_g$ orbitals in the large-*U* limit, as reported in the online supplementary material. This $e_g$-orbital degenerate Mott insulator has been discussed with equal importance of spin and orbital degrees of freedom [48,50,51]. In La$_3$Ni$_2$O$_7$, the molecular energy $\epsilon _{x+}$ is small and the introduction of a small number of $|x,+\rangle _\sigma$ will cost molecular energy but gain kinetic energy. Similar to the second scenario mentioned in the single-orbital bilayer Hubbard model in Fig. 1b2, a small number of $|x,+\rangle _\sigma$ will introduce the self-doping effect that generates the doublon state $|x,-\rangle _\sigma \otimes |x,+\rangle _\sigma$ together with the self-doped holes, as depicted in Fig. 3b. Therefore, we propose that the effective theory of the La$_3$Ni$_2$O$_7$ system is described by the two-orbital self-doped molecular Mott insulator.

The low-energy effective Hamiltonian in the large-*U* limit can be derived following the work of Castellani *et al.* [50]. Because the bonding orbitals $|x,+\rangle _\sigma$ are weakly correlated, its effects can be well approximated by doping additional holes into the molecular Mott insulator described by $H_A$. At $J_H=0$, the Hubbard interaction associated with the anti-symmetric molecular orbitals $U_A^{xz}=U_A^{xx}=U_A^{zz}=U_A$. We define spin operator $\boldsymbol {S}_{i\alpha \beta }=\frac{1}{2} c_{i,\alpha -}^\dagger \boldsymbol {\sigma } c_{i,\beta -}$ and the density operator $n_{i\alpha \beta }=c_{i,\alpha -}^\dagger c_{i,\beta -}$, with $c_{i,\beta -}=(c_{i,\beta -,\uparrow }, \, c_{i,\beta -,\downarrow })^T$. Note that in the case with $\alpha =\beta$, $\boldsymbol {S}_{i\alpha \alpha }$ and $n_{i\alpha \alpha }$ are respectively just the spin and density operators associated with the electrons in the same anti-symmetric orbital $\alpha -$. The effective interaction Hamiltonian derived from $H_A$ can be written as


(8) \begin{eqnarray*}
H_{A,int}&=& \frac{4}{U_A}\sum _{\langle ij\rangle }\sum _{\alpha \alpha ^\prime \beta \beta ^\prime } \\
&&\bigg [t_{ij}^{\alpha \beta }t_{ij}^{\alpha ^\prime \beta ^\prime }\boldsymbol {S}_{i\alpha \alpha ^\prime } \cdot \boldsymbol {S}_{j\beta ^\prime \beta }
- \frac{1}{4} \Big[t_{ij}^{\alpha \beta }t_{ij}^{\alpha ^\prime \beta ^\prime } - t_{ij}^{\alpha \bar{\beta ^\prime }}t_{ij}^{\alpha ^\prime \bar{\beta }} (-1)^{\delta _{\beta \beta ^\prime }} \\
&& - t_{ij}^{\bar{\alpha }\beta ^\prime }t_{ij}^{\bar{\alpha ^\prime }\beta }(-1)^{\delta _{\alpha \alpha ^\prime }}\Big ]n_{i\alpha \alpha ^\prime } n_{j \beta ^\prime \beta }\bigg ].
\end{eqnarray*}

Here the orbital index can take either *x* or *z*, referring to the two anti-symmetric orbitals $|x,-\rangle$ and $|z,-\rangle$. The bar notation, e.g. $\bar{\beta }$, refers to the other orbital. This Hamiltonian is equivalent to the Kugel–Khomskii (KK) Hamiltonian [48] in the limit $J_H=0$. At one electron per site, i.e. without hole doping, the parent phase of the above Hamiltonian is a ferro-orbital order in the two molecular orbitals with $\boldsymbol {Q}=(\pi ,\pi )$ spin anti-ferromagnetism, similar to the case of KCuF$_3$ [49].

In the self-doping regime, the holes are introduced by electrons transferring to the $|x,+\rangle$ orbital, resulting in the hole density $n_h=n_{x+}$. More holes can be introduced through external chemical doping so that $n_h>n_{x+}$. To provide a physical picture, we focus on the self-doping regime in the following discussions. It is essential to elucidate the correlation effect of different types of particles appropriate to the La$_3$Ni$_2$O$_7$ system. With one electron per site, the $|x,-\rangle _\sigma$ and $|z,-\rangle _\sigma$ electrons can only move to neighboring empty sites, which is of density ${\sim }n_{x+}/2\ll 1$. Therefore, electrons occupying the two anti-symmetric orbitals are highly correlated. The symmetric electrons $|x,+\rangle _\sigma$, on the other hand, can hop to the neighboring anti-symmetric $|x,-\rangle _\sigma$ orbital of the same spin, with density ${\sim } n_{x-}/2\sim 1/2$, and are hence weakly correlated. We expect that the superconductivity is primarily induced by the $|x,-\rangle _\sigma$ and $|z,-\rangle _\sigma$ electrons.

The in-plane hopping of the two anti-symmetric orbitals is strongly renormalized due to the correlation effect. The hopping part in the large-*U* limit follows from the first term in $H_A$:


(9) \begin{eqnarray*}
H_{A,t}=-\sum _{\langle ij\rangle ,\alpha \alpha ^\prime }t_{ij}^{\alpha \alpha ^\prime } \hat{P}_G \left(c_{i,\alpha -,\sigma }^\dagger c_{j,\alpha ^\prime -,\sigma }+\text{H.c.}\right)\hat{P}_G.
\end{eqnarray*}

Here $\hat{P}_G$ denotes the projection onto the low-energy Hilbert space in the large-*U* limit. The molecular hopping parameters $t_{ij}^{\alpha \alpha ^\prime }$ can be obtained from first-principle calculations [27,46].

The superconducting pairing symmetry is analyzed using renormalized mean field theory [41]. The effective Hamiltonian $H_{\text{eff}}$ of the self-doped molecular Mott insulator is given by


(10) \begin{eqnarray*}
H_{\text{eff}}=H_{A,t}+H_{A,int},
\end{eqnarray*}

where we set $t_{ij}^+$ in $H_S$ equals $t_{ij}^{xx}$ in $H_{A,t}$. This is a two-orbital *t*-*J* model, where $H_{A,t}$ is the kinetic energy term, describing a renormalized band of anti-symmetric orbitals whose Fermi surface is similar to that given by DFT calculations ($\beta$ band in [46]). In the self-doping regime, the number of self-doped holes $n_h$ is controlled by $\epsilon _{x+}$ in $H_S$ with $n_h=n_{x+}$. To account for the double occupancy constraint imposed by $\hat{P}_G$ in $H_{A,t}$, we introduce the Gutzwiller factor $g_{t}^{\alpha \alpha ^\prime }={2n_h}/{\sqrt{(2-n_{\alpha -})(2-n_{\alpha ^\prime -})}}$ [52,53], where $n_h$ denotes the number of self-doped holes, and $n_{\alpha -}$ with $\alpha =x,z$ is the total electron number associated with the molecular orbital.

The pairing mean field in the singlet channel is defined as


(11) \begin{eqnarray*}
\Delta _{ij}^{\alpha \alpha ^\prime }=\left\langle c_{i,\alpha -,\uparrow }^\dagger c_{j,\alpha ^\prime -,\downarrow }^\dagger \right\rangle -\left\langle c_{i,\alpha -,\downarrow }^\dagger c_{j,\alpha ^\prime -,\uparrow }^\dagger \right\rangle
\end{eqnarray*}

with $\alpha ,\alpha ^\prime \in \lbrace x,z\rbrace$. The pairing therefore contains contributions from both the inter- and intra-orbital processes. The mean field Hamiltonian and the self-consistent equations are derived in the online supplementary material. In comparison to the single orbital *t*-*J* model, there are two types of nearest-neighbor pairing order parameters: the intra-orbital pairing $\Delta ^{xx},\, \Delta ^{zz}$ and the inter-orbital pairing $\Delta ^{xz}$, and their competition leads to different pairing symmetries.

**Figure 4.**
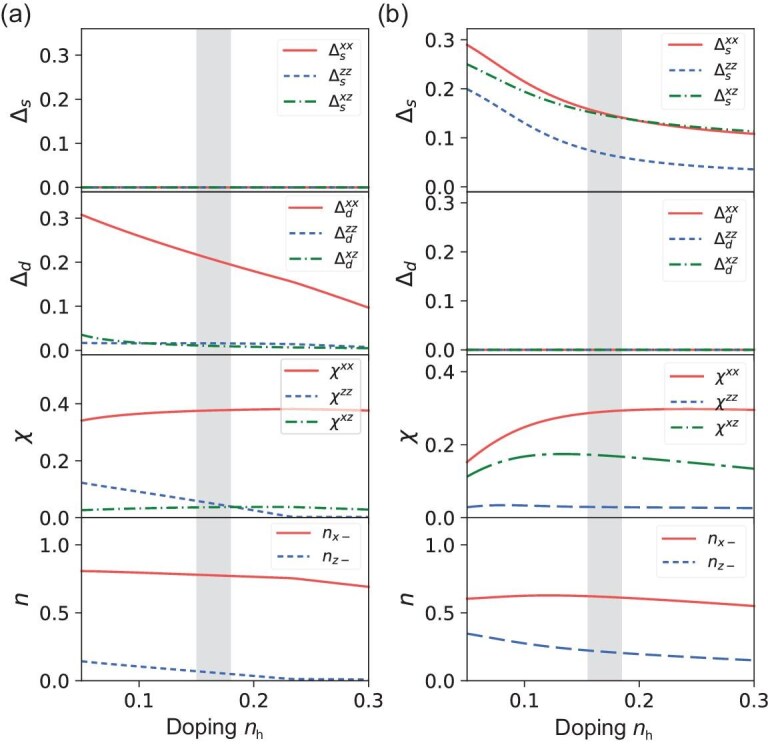
Mean field results for the two-orbital self-doped molecular Mott insulator obtained with in-plane intra-orbital hoppings $t^{xx}=1$, $t^{zz}=0.2$, and inter-orbital hopping $t^{xz}=0.1$ (a), $t^{xz}=0.6$ (b), plotted as a function of the self-doped hole density $n_h$. The Hubbard interaction is set as $U_A=6$. The figures from top to bottom show the results for the pairing component in the extended *s*-wave $\Delta _{s}$ and *d*-wave $\Delta _{d}$ channels, the hopping mean field $\chi$ and the occupation numbers $n_{x-}$, $n_{z-}$. With increasing inter-orbital hybridization $t^{xz}$, we find that the pairing symmetry changes from *d*-wave to *s*-wave. We estimate from DFT calculations [46] that $t^{xz}\approx 0.4$–0.6 and self-doping $n_h\approx 0.17$ (area shaded in gray), corresponding to the $\alpha$ band.

The parameters in the calculations are chosen as follows. The Hubbard interaction $U_A=6$ and the nearest-neighbor hoppings are set as $t^{xx}=1,\, t^{zz}=0.2$. As for inter-orbital hoppings, we consider two cases with $t^{xz}=0.1$ and $t^{xz}=0.6$. The calculations are performed as a function of the self-doped hole density $n_h$. Each $n_h$ corresponds to a specific $\epsilon _{x+}$, e.g. $n_h=0.1$ can be obtained with $\epsilon _{x+}\approx 3$. The pairing symmetry can be categorized according to the representation of the lattice symmetry group $D_{4h}$. In the singlet channel, pairing between the nearest neighbors can belong to either the extended *s*-wave ($A_{1g}$ representation), denoted as $\Delta _s$, with the same sign in the *x* and *y* directions, or the *d*-wave ($B_{1g}$ representation), denoted as $\Delta _d$, with alternating signs. The mean field results are plotted in Fig. 4. For $t^{xz}=0.1$, as shown in Fig. 4a, we find that the pairing symmetry agrees with the *d*-wave, similar to the single-orbital *t*-*J* model. In this case, the pairings are found to be dominated by $\Delta ^{xx}$ (red solid line), i.e. the intra-orbital pairing within the $|x,-\rangle$ orbital. We find that $\Delta ^{xz}$ (green dash–dot line) is significantly smaller; $\Delta ^{zz}$ is also small due to a small particle number $n_z$, as shown in the lowest panel. For $t^{xz}=0.6$, as shown in Fig. 4b, the pairing solution is found to agree with the extended *s*-wave. In this case, both intra- and inter-orbital pairings are of similar magnitude, suggesting strong inter-orbital effects. Moreover, the electron occupation number difference $n_{x-}-n_{z-}$ becomes much smaller as the inter-orbital hopping increases from $t^{xz}=0.1$ to $t^{xz}=0.6$, which also points to enhanced inter-orbital effects. We therefore conclude based on the above calculations that the extended *s*-wave pairing can be favored due to a significant inter-orbital effect. For an intermediate value of $t^{xz}$, the competition between *s*- and *d*-wave instability requires further study.

### Effect of *U* and $J_H$ on the molecule state

In the previous section we showed that, due to the crystal field splitting and Jahn–Teller effect, the two sets of $e_g$ orbitals are relevant for the low-energy physics of La$_3$Ni$_2$O$_7$. The inter-layer hoppings $t_\perp ^{x,z}$ further introduce the molecular energy splitting between the inter-layer (anti-)symmetric molecular orbitals. The effective low-energy theory in the large-*U* limit is derived based on the local electronic structure. In this section, we demonstrate that the low-energy orbitals we identified play the dominant role in the low-energy physics by performing exact diagonalization on a local molecular system composed of two Ni$^{2.5+}$ ions, one from the top layer and the other from the bottom layer. We analyze the impact of the finite Hubbard repulsion *U* and the role of inter-layer hopping—especially the $t_\perp ^x$ term—and we examine the influence of Hund’s coupling $J_H$. Consider a single Ni$_2^{5+}$ molecule with a total of three electrons occupying eight orbitals formed by the two sets of $e_g$ orbitals. The Hamiltonian for such a molecule can be written as


**Figure 5.**
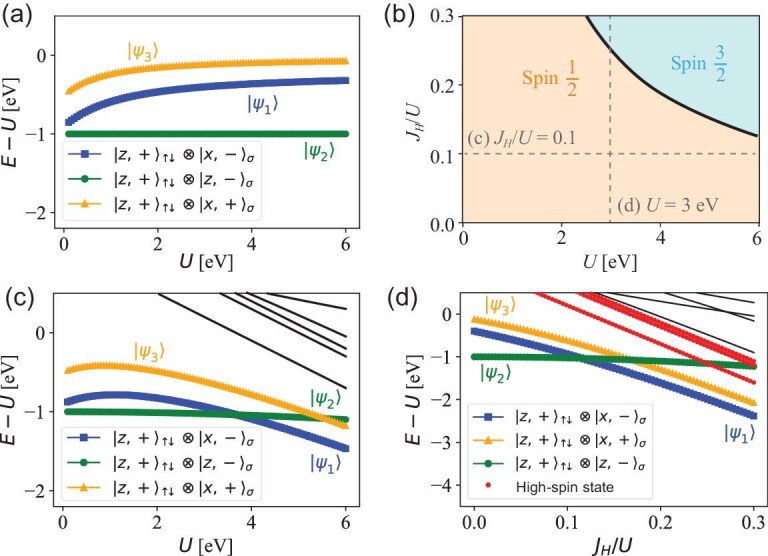
Local spectra and projected wave-function components of the low-energy eigenstates. (a) The spectra are obtained under $t_\perp ^z=-1\,\text{eV}$, $t_\perp ^x=0.2\,\text{eV}$, $E_x=1.3\,\text{eV}$, $J_H=0\,\text{eV}$. The dominant wave-function components are plotted along the spectra of the three lowest-energy eigenstates according to $|z,+\rangle _{\uparrow \downarrow } \otimes |x,-\rangle$ (blue), $|z,+\rangle _{\uparrow \downarrow } \otimes |z,-\rangle$ (green) and $|z,+\rangle _{\uparrow \downarrow } \otimes |x,+\rangle$ (orange). The first two electronic configurations correspond to the two anti-symmetric orbitals forming the molecular Mott insulator, and the last one contributes to the self-doping effect. (b) The *U*-$J_H/U$ phase diagram obtained by analyzing the single-site spectra. The parameters $t_\perp ^{x,z}, E_x$ are the same as in (a). In the region marked by spin $1/2$, the low-energy subspace is spanned by the three spin-$1/2$ states $|\psi _{1,2,3}\rangle$. With increasing $J_H$, the spin-$3/2$ state becomes energetically favored over one of the three spin-$1/2$ states, as marked by the phase boundary. The two dashed lines denote $J_H/U=0.1$ and $U=3$ eV, respectively, with corresponding energy spectra plotted in (c) and (d). (c) The spectra obtained with $J_H/U=0.1$. The two eigenstates with electrons occupying different orbitals have smaller slope in *U* due to Hund’s coupling. Around $U=4$ eV, the local electronic spectra contain two nearly degenerate anti-symmetric orbitals $|x,-\rangle$ and $ |z,-\rangle$, with $ |x,+\rangle$ above by ${\sim } t_\perp ^x$. (d) The spectra obtained with $U=3$ eV as a function of $J_H/U$. The molecular Mott insulating regime is valid below $J_H/U\sim 0.25$. With larger $J_H/U$, the high-spin states become energetically favored over the $|\psi _2\rangle$.

(12) \begin{eqnarray*}
H_{\text{molecule}}&=& \sum _{\alpha \sigma } t_\perp ^\alpha \Big(c_{t\alpha \sigma }^\dagger c_{b\alpha \sigma } + \text{H.c.}\Big)+ E_x\sum _{l\sigma } n_{x\sigma } \\
&& +\, U\sum _{l\alpha }n_{l\alpha \uparrow }n_{l\alpha \downarrow }+U^{\prime }\sum _l n_{lx}n_{lz} \\
&& +\, J_H\sum _l\sum _{\sigma \sigma ^{\prime }}c_{lx\sigma }^\dagger c_{lz\sigma ^{\prime }}^\dagger c_{lx\sigma ^{\prime }}c_{lz\sigma } \\
&& +\, J_P\sum _l\sum _\alpha c_{l\alpha \uparrow }^\dagger c_{l\alpha \downarrow }^\dagger c_{l\bar{\alpha }\downarrow }c_{l\bar{\alpha }\uparrow },
\end{eqnarray*}

where we set $E_z=0$ for convenience. In the interacting part, $J_H=J_P=J$ denotes Hund’s coupling and the invariance under symmetry operations yields $U^{\prime }=U-2J$. The hopping and interaction parameters here are associated with the atomic orbitals. The energy spectra are obtained through exact diagonalization of $H_{\text{molecule}}$ and the wave-function contents of the eigenstates are analyzed, as shown in Fig. 5. The parameters here are $t_\perp ^z=-1$ eV and $E_x=1.3$ eV, as estimated from the DFT calculations in [46] with a slightly larger $t^x_\perp =0.2$ eV.

For $J_H=0$, as depicted in Fig. 5a, the low-energy space contains three eigenstates: $\lbrace |\psi _1\rangle ,\, |\psi _2\rangle ,\, |\psi _3\rangle \rbrace$. The eigenstates are usually found to be a hybridization of multiple electronic configurations. For the three lowest eigenstates, we find that $|\psi _1\rangle$ is dominated by $|z,+\rangle _{\uparrow \downarrow }\otimes |x,-\rangle _\sigma$ (blue), $|\psi _2\rangle$ is strictly $|z,+\rangle _{\uparrow \downarrow }\otimes |z,-\rangle _\sigma$ (green) and $|\psi _3\rangle$ is dominated by $|z,+\rangle _{\uparrow \downarrow }\otimes |x,+\rangle _\sigma$ (yellow). In particular, states $|\psi _{1,2,3}\rangle$ overlap with the corresponding dominating states by $\lbrace {\sim }0.86, 1, {\sim }0.85\rbrace$ in the large-*U* limit. As revealed by the dominating wave-function components, the $|z,+\rangle$ orbital remains doubly occupied, in agreement with the previous analysis that $|z,+\rangle$ lies well below other orbitals. Furthermore, at $U=0$, the two anti-symmetric orbitals, i.e. $|x,-\rangle$ and $|z,-\rangle$, are nearly degenerate, as reflected by states $|\psi _{1,2}\rangle$ being close in the spectra. The $|x,+\rangle$ orbital lies above by $t_{\perp }^x$, leading to the self-doping effect. The self-doping effect can be weakened with increasing $t_{\perp }^x$ because the molecular gap between $|\psi _1\rangle$ and $|\psi _3\rangle$ will become larger. As *U* increases, the energy of the $|\psi _1\rangle$ and $|\psi _3\rangle$ orbitals slightly increases due to the hybridization with other orbitals.

The effects of $J_H$ are also of interest, as studied in various works [28,54]. To demonstrate the effects of Hund’s coupling in the proposed molecular Mott insulator regime, we perform systematic calculations under different *U* and $J_H/U$. The low-energy space is analyzed; see the phase diagram depicted in Fig. 5b. For small $J_H/U$, the low-energy space is spanned by the three spin-$1/2$ states, namely, $|\psi _{1,2,3}\rangle$, in agreement with the proposed regime for the self-doped molecular Mott insulator. As $J_H$ becomes larger, the high-spin states become energetically favored and the low-energy space becomes more complicated. The low-energy spectra along the two dashed lines in Fig. 5b are shown in Fig. 5c and d, respectively. Firstly, as depicted in Fig. 5c, the relative strength of Hund’s coupling is fixed at $J_H=0.1U$ and the local electronic spectra is solved as a function of *U*. The low-energy space is similar to the previous two cases. However, the two states with electrons occupying different orbitals, i.e. $|\psi _1\rangle$ and $|\psi _3\rangle$, are favored by Hund’s coupling in the large-*U* regime. In particular, for $U\sim 4$ eV according to the estimation of La$_3$Ni$_2$O$_7$ from first-principle calculations [47], the electronic structure is in exact agreement with the proposed self-doped molecular Mott insulator. Next, we fix the value of $U=3$ eV and study the energy spectra as a function of $J_H/U$. As shown in Fig. 5d, we find that the molecular Mott insulator scenario is valid for $J_H/U\lesssim 0.25$. As $J_H/U$ increases, the high-spin states (red) become energetically favored over $|\psi _2\rangle$ (green).

### CONCLUSION

In this paper, we start with two $e_g$ orbitals ($d_{x^2-y^2}$ and $d_{z^2}$) for double layer La$_3$Ni$_2$O$_7$. We suppose that the inter-layer coupling for $d_{z^2}$ orbitals is strong and that the two Ni sites in the top and bottom layers form a molecule. The bonding states of $d_{z^2}$ are all occupied, leaving one $e_g$ electron per molecule on average for the anti-bonding $d_{z^2}$ and both bonding and anti-bonding states of $d_{x^2-y^2}$. In the large limit of on-site Coulomb repulsion *U*, the system is described by the KK model or molecular Mott insulator with two orbitals (anti-bonding $d_{z^2}$ and bonding $d_{x^2-y^2}$) if all the anti-bonding states of $d_{x^2-y^2}$ are empty, and the system is described by the self-doped molecular Mott insulator if the anti-bonding $d_{x^2-y^2}$ is slightly occupied. The latter may form a molecular doublon with the bonding $d_{x^2-y^2}$ orbital of the same spin, which does not cost *U*, and hence provides holes in the background of the Mott insulator. We argue that the low-energy physics is given by the doped two orbital *t*-*J* model (more precisely, the KK model) on a square lattice. Superconductivity arises from nearest-neighbor orbital and spin coupling, whose pairing symmetry depends on the inter-orbital pairing strength. The effects of Hund’s coupling is further analyzed based on the exact diagonalization calculations, and the self-doped molecular Mott insulating regime is found to be valid under the realistic estimation of the model parameters. Our theory predicts more complex spin excitation in La$_3$Ni$_2$O$_7$ than in cuprates because of the two orbitals. Chemical hole doping, such as partial replacement of La by valence $2+$ ions (Ca or Sr) and interstitial oxygens, could introduce additional mobile holes and hence is good for superconductivity. For electron doping, the apical oxygen vacancies between bilayer Ni atoms destroy the molecular orbital, especially the anti-binding Fermi surface [46]. Therefore, the oxygen vacancies play a similar role as impurities on CuO$_2$ planes in cuprates and severely damage the superconductivity.

## Supplementary Material

nwaf353_Supplemental_File
