# Supplementary material for: Self-doped molecular Mott insulator for bilayer high-temperature superconducting La3Ni2O7
Source: Natl Sci Rev. 2025 Aug 22;12(10):nwaf353. doi: 10.1093/nsr/nwaf353 (PMC12512141; doi:10.1093/nsr/nwaf353)
Supplement: nwaf353_Supplemental_File [file nwaf353_supplemental_file.pdf]

# Supplemental Material for "Self-doped Molecular Mott Insulator for Bilayer High-Temperature Superconductivity"

## LOCAL ELECTRONIC STRUCTURE IN THE $U \to \infty$ LIMIT

To analyze the local electronic structure, we consider a 2-site molecule formed by $Ni_2^{5+}$ in the $U \to \infty$ limit. The local Hamiltonian of the molecule reads:

$$H_0 = \sum_{\alpha\sigma} t_\perp^\alpha \left( c_{t\alpha\sigma}^\dagger c_{b\alpha\sigma} + \text{h.c.} \right) + \sum_{l=t,b} \sum_{\alpha} U n_{l\alpha\uparrow} n_{l\alpha\downarrow} + \sum_{l=t,b} U' n_{lx} n_{lz} + H_{onsite}. \tag{S1}$$

For consistency, we use $l = t, b$ to denote the two $Ni^{2.5+}$ ions from the two layers. The interlayer hybridization amplitude is denoted by $t_\perp^\alpha$, with $\alpha = x, z$ for the atomic orbital index. Note there is no inter-orbital hybridization due to the symmetry of the atomic orbital wave functions. We expect $|t_\perp^z| > |t_\perp^x|$ due to the significant wave function overlap between Ni $3d_{z^2}$ orbitals and the interlayer oxygen $p_z$ orbitals. As mentioned in the main text, one will find $t_\perp^z < 0$ from the sign of the wave functions. For simplicity, we ignore the Hund's coupling and set $U = U'$.

In addition, the Jahn-Teller effect is described by the orbital onsite energy

$$H_{onsite} = E_x \sum_{l\sigma} n_{x\sigma} + E_z \sum_{l\sigma} n_{z\sigma}. \tag{S2}$$

Here $E_x > 0$ and $E_z = 0$ due to elongated apical oxygen bonds in the Ni-O octahedron.

For such a molecule, there are in total 3 electrons occupying the 8 local energy levels formed by the two sets of $e_g$ orbitals of the $Ni_2^{5+}$ ions. Therefore there are in total 56 possible states. We begin by noting the symmetry and conserved quantities in $H_0$ in the local orbitals. We define the total spin polarization $s_z$ as the total spin along the $z$ direction and the orbital polarization $\tau_z = -1/2(+1/2)$ for $z(x)$ orbital, respectively. The operators are defined as:

$$\hat{s}_z = \frac{1}{2} \sum_{l,\alpha} (n_{l\alpha\uparrow} - n_{l\alpha\downarrow}); \tag{S3}$$

$$\hat{\tau}_z = \frac{1}{2} \sum_{l,\sigma} (n_{lx\sigma} - n_{lz\sigma}); \tag{S4}$$

$$\hat{s}_z \hat{\tau}_z = \frac{1}{2} \sum_{l} (n_{lx\uparrow} - n_{lx\downarrow} - n_{lz\uparrow} + n_{lz\downarrow}) \tag{S5}$$

In the last line, we introduce a composite operator $\hat{s}_z\hat{\tau}_z$, describing the spin-orbital locking. All 3 operators commute with $H_0$ and give good quantum numbers denoted by $s_z, \tau_z, s_z\tau_z$, respectively.

We consider the local electronic states in the large $U$ limit and treat interlayer hopping in $H_\perp$ as small parameter. There are in total 56 possible states, and 8 of them contains 3 electrons in the same layer, leading to total Hubbard repulsion of $3U$. The remaining 48 states contains 2 electrons in one layer and 1 electron in the other layer, which corresponds to energy cost from Hubbard repulsion of $U$. In the low-energy limit, we consider the latter 48 states and set $E_0 = U$ as the energy reference point.

The 48 states can be further categorized according to the three good quantum numbers $(s_z, \tau_z, s_z\tau_z)$ into 4 groups and we calculate their energy eigen value resulting from interlayer hybridization $t_\perp^z$ ($t_\perp^x$ is set to 0 here for simplicity).

Group I: $(s_z = \pm 3/2, \tau_z = \pm 1/2, s_z\tau_z = \pm 1/2)$. In this case, the three electrons are of the same spin and occupy 3 out of 4 possible orbitals from the two layers, leading to 8 states in total. The energy eigen values are $\pm t_\perp^z$ for $\tau_z = 1/2$ (each is two-fold degenerate) and 0 for $\tau_z = -1/2$ (four-fold degenerate).

Group II: $(s_z = \pm 1/2, \tau_z = \pm 3/2, s_z\tau_z = \pm 1/2)$. In this case, the three electrons are orbital-polarized. There are 8 states in total. The energy eigen values are $\pm t_\perp^z$ for $\tau_z = -3/2$ (each is two-fold degenerate) and 0 for $\tau_z = 3/2$ (four-fold degenerate).

Group III: $(s_z = \pm 1/2, \tau_z = \pm 1/2, s_z\tau_z = \pm 3/2)$. In this case, the spin and orbital are locked, e.g the case with all spin-up electrons in $x$ orbital and all spin-down electrons in $z$ orbital corresponds to $s_z\tau_z = 3/2$. There are in total 8 states belonging to this sector. The energy eigen values are $\pm t_\perp^z$ for $\tau_z = 1/2$ (each is two-fold degenerate) and 0 for $\tau_z = -1/2$ (four-fold degenerate).

Group IV: $(s_z = \pm 1/2, \tau_z = \pm 1/2, s_z\tau_z = \pm 1/2)$. Here none of the three conserved quantities are fully-polarized. There are 24 states belonging to this group. The 24 states can be further divided into 4 subgroups which are labeled as $(s_z, \tau_z, s_z\tau_z) \in$

$\Big\{(\frac{1}{2},-\frac{1}{2},\frac{1}{2}),(-\frac{1}{2},-\frac{1}{2},-\frac{1}{2}),(\frac{1}{2},\frac{1}{2},-\frac{1}{2}),(-\frac{1}{2},\frac{1}{2},\frac{1}{2})\Big\}$. Take the first subgroup as an example, the 6 states can be denoted as:

$$
\begin{aligned}
|\psi_1\rangle &= c^\dagger_{bx\uparrow}c^\dagger_{tz\uparrow}c^\dagger_{tz\downarrow}|\mathrm{vac}\rangle, \quad |\psi_2\rangle = c^\dagger_{tx\uparrow}c^\dagger_{bz\uparrow}c^\dagger_{bz\downarrow}|\mathrm{vac}\rangle,\\
|\psi_3\rangle &= c^\dagger_{bx\uparrow}c^\dagger_{tz\uparrow}c^\dagger_{bz\downarrow}|\mathrm{vac}\rangle, \quad |\psi_4\rangle = c^\dagger_{tx\uparrow}c^\dagger_{tz\uparrow}c^\dagger_{bz\downarrow}|\mathrm{vac}\rangle,\\
|\psi_5\rangle &= c^\dagger_{bx\uparrow}c^\dagger_{tz\downarrow}c^\dagger_{bz\uparrow}|\mathrm{vac}\rangle, \quad |\psi_6\rangle = c^\dagger_{tx\uparrow}c^\dagger_{tz\downarrow}c^\dagger_{bz\uparrow}|\mathrm{vac}\rangle.
\end{aligned}
$$

In writing the interlayer hybridization, we choose the following basis for convenience:

$$
\begin{aligned}
|1\rangle &= \frac{1}{\sqrt{2}}(c^\dagger_{bx\uparrow}c^\dagger_{tz\uparrow}c^\dagger_{tz\downarrow} + c^\dagger_{tx\uparrow}c^\dagger_{bz\uparrow}c^\dagger_{bz\downarrow})|\mathrm{vac}\rangle,\\
|2\rangle &= \frac{1}{\sqrt{2}}(c^\dagger_{bx\uparrow}c^\dagger_{tz\uparrow}c^\dagger_{tz\downarrow} - c^\dagger_{tx\uparrow}c^\dagger_{bz\uparrow}c^\dagger_{bz\downarrow})|\mathrm{vac}\rangle,\\
|3\rangle &= \frac{1}{2}(c^\dagger_{bx\uparrow} + c^\dagger_{tx\uparrow})(c^\dagger_{tz\uparrow}c^\dagger_{bz\downarrow} - c^\dagger_{tz\downarrow}c^\dagger_{bz\uparrow})|\mathrm{vac}\rangle,\\
|4\rangle &= \frac{1}{2}(c^\dagger_{bx\uparrow} - c^\dagger_{tx\uparrow})(c^\dagger_{tz\uparrow}c^\dagger_{bz\downarrow} - c^\dagger_{tz\downarrow}c^\dagger_{bz\uparrow})|\mathrm{vac}\rangle\\
|5\rangle &= \frac{1}{2}(c^\dagger_{bx\uparrow} + c^\dagger_{tx\uparrow})(c^\dagger_{tz\uparrow}c^\dagger_{bz\downarrow} + c^\dagger_{tz\downarrow}c^\dagger_{bz\uparrow})|\mathrm{vac}\rangle,\\
|6\rangle &= \frac{1}{2}(c^\dagger_{bx\uparrow} - c^\dagger_{tx\uparrow})(c^\dagger_{tz\uparrow}c^\dagger_{bz\downarrow} + c^\dagger_{tz\downarrow}c^\dagger_{bz\uparrow})|\mathrm{vac}\rangle
\end{aligned}
\tag{S6}
$$

Note the states $|5\rangle$ and $|6\rangle$ don't hybridize with other states in this subgroup under interlayer hopping $t^\alpha_\perp$ and their energy eigen values are given by $E_5 = t^x_\perp$, $E_6 = -t^x_\perp$. Under the basis of the first 4 states, the Hamiltonian associated with $t^\alpha_\perp$ can be written as:

$$
H_\perp = \begin{pmatrix} 0 & 0 & \sqrt{2}t^z_\perp & 0 \\ 0 & 0 & 0 & \sqrt{2}t^z_\perp \\ \sqrt{2}t^z_\perp & 0 & t^x_\perp & 0 \\ 0 & \sqrt{2}t^z_\perp & 0 & -t^x_\perp \end{pmatrix}. \tag{S7}
$$

For $t^x_\perp = 0$, this subgroup has the lowest energy eigen value $-\sqrt{2}t^z_\perp$, which is lower than the minimum of other groups $-t^z_\perp$. With a nonzero $t^x_\perp$, the energy eigen value can be obtained as:

$$
E^\pm_1 = \pm\frac{1}{2}\Big(t^x_\perp + \sqrt{(t^x_\perp)^2 + 8(t^z_\perp)^2}\Big), \tag{S8}
$$

$$
E^\pm_2 = \pm\frac{1}{2}\Big(t^x_\perp - \sqrt{(t^x_\perp)^2 + 8(t^z_\perp)^2}\Big). \tag{S9}
$$

Consider the case with $E_x - E_z \approx |t^z_\perp|$ and $t^z_\perp < 0$ as mentioned in the main text, the low-energy space is spanned by the following three states:

$$
\begin{aligned}
|\psi_1\rangle &= a_1|z,+\rangle_{\uparrow\downarrow}\otimes|x,-\rangle_\sigma + \text{other components}, \quad \lambda_1 = -\frac{1}{2}\Big(t^x_\perp + \sqrt{(t^x_\perp)^2 + 8(t^z_\perp)^2}\Big) + E_x,\\
|\psi_2\rangle &= |z,+\rangle_{\uparrow\downarrow}\otimes|z,-\rangle_\sigma, \quad \lambda_2 = t^z_\perp,\\
|\psi_3\rangle &= a_3|z,+\rangle_{\uparrow\downarrow}\otimes|x,+\rangle_\sigma + \text{other components}, \quad \lambda_3 = \frac{1}{2}\Big(t^x_\perp - \sqrt{(t^x_\perp)^2 + 8(t^z_\perp)^2}\Big) + E_x.
\end{aligned}
$$

Here $\lambda$ denotes the energy eigen value of $H_0$ with respect to the reference point $E_0 = U$. For $E_x = 0.8$, $t^z_\perp = -1$ and $t^x_\perp = 0.2$, the three eigen energies are given by $\{\lambda_1 = -0.72,\ \lambda_2 = -1,\ \lambda_3 = -0.52\}$. The state $|z,+\rangle_{\uparrow\downarrow}\otimes|z,-\rangle_\sigma$ is exactly the eigenstate $|\psi_2\rangle$. The other two eigenstates $|\psi_1\rangle$ and $|\psi_3\rangle$ are dominated by their first components with $a_1 \approx 0.86$ and $a_3 \approx 0.85$.

## RENORMALIZED MEAN FIELD CALCULATION

The effective model in given by eq. (10) in the main text:

$$H_{\text{eff}} = H_S + H_{A,t} + H_{A,int} \tag{S10}$$

The hopping part of the bonding electrons from $|x,+\rangle$ orbital is described by eq. (6):

$$H_S = -\sum_{\langle ij\rangle,\sigma} t^{+}_{ij}(c^{\dagger}_{i,x+,\sigma}c_{j,x+,\sigma} + \text{h.c.}) + \epsilon_{x+}\sum_{i\sigma} c^{\dagger}_{i,x+,\sigma}c_{i,x+,\sigma}, \tag{S11}$$

where we use $c^{\dagger}_{i,x+,\sigma}$ to denote the creation operator corresponding to $|x,+\rangle_{\sigma}$ with spin $\sigma$ and $\epsilon_{x+} > 0$ denotes the molecular energy shift of the bonding orbital.

The hopping part of the antisymmetric electrons from $|x,-\rangle$ and $|z,-\rangle$ orbitals is given by eq. (9):

$$\begin{aligned} H_{A,t} &= -\sum_{\langle ij\rangle,\alpha\alpha'} t^{\alpha\alpha'}_{ij}\hat{P}_G(c^{\dagger}_{i,\alpha-,\sigma}c_{j,\alpha'-,\sigma} + \text{h.c.})\hat{P}_G \\ &= -\sum_{\langle ij\rangle,\alpha\alpha'} g^{\alpha\alpha'}_t t^{\alpha\alpha'}_{ij}(c^{\dagger}_{i,\alpha-,\sigma}c_{j,\alpha'-,\sigma} + \text{h.c.}) \end{aligned} \tag{S12}$$

Here $c^{\dagger}_{i,\alpha-,\sigma}$ with $\alpha,\alpha' = x,z$ is the creation operator associated with $|x,-\rangle$ and $|z,-\rangle$. $\hat{P}_G$ in the first line denotes the projection operation from the double-occupancy constraint in the large-$U$ limit. The Gutzwiller factor is defined as: $g^{\alpha\alpha'}_t = \frac{2n_h}{\sqrt{(2-n_{\alpha-})(2-n_{\alpha'-})}}$, where $n_h = n_{x+}$ denotes the number of self-doped holes, and $n_{\alpha-}$ is the total electron number from the orbital $|x,-\rangle$ and $|z,-\rangle$.

The effective interaction term of the antisymmetric electrons is derived in eq. (8):

$$H_{A,int} = \frac{4}{U_A}\sum_{\langle ij\rangle}\sum_{\alpha\alpha'\beta\beta'}\left[t^{\alpha\beta}_{ij}t^{\alpha'\beta'}_{ij}\boldsymbol{S}_{i\alpha\alpha'}\cdot\boldsymbol{S}_{j\beta'\beta} - \frac{1}{4}\left(t^{\alpha\beta}_{ij}t^{\alpha'\beta'}_{ij} - t^{\alpha\bar{\beta}'}_{ij}t^{\alpha'\bar{\beta}}_{ij}(-1)^{\delta_{\beta\beta'}} - t^{\bar{\alpha}\beta'}_{ij}t^{\bar{\alpha}'\beta}_{ij}(-1)^{\delta_{\alpha\alpha'}}\right)n_{i\alpha\alpha'}n_{j\beta'\beta}\right]. \tag{S13}$$

The spin operator is defined as $\boldsymbol{S}_{i\alpha\beta} = \frac{1}{2}c^{\dagger}_{i,\alpha-}\boldsymbol{\sigma}c_{i,\beta-}$ and the density operator $n_{i\alpha\beta} = c^{\dagger}_{i,\alpha-}c_{i,\beta-}$, with $c_{i,\beta-} = (c_{i,\beta-,\uparrow}, c_{i,\beta-,\downarrow})^T$. To decouple the two quartic terms in the interaction Hamiltonian, we introduce two types of mean fields. One is the singlet pairing mean field $\Delta^{\alpha\alpha'}_{ij}$ as in eq. (11):

$$\Delta^{\alpha\alpha'}_{ij} = \langle c^{\dagger}_{i,\alpha-,\uparrow}c^{\dagger}_{j,\alpha'-,\downarrow}\rangle - \langle c^{\dagger}_{i,\alpha-,\downarrow}c^{\dagger}_{j,\alpha'-,\uparrow}\rangle. \tag{S14}$$

The other one is the hopping mean field $\chi^{\alpha\alpha'}_{ij}$:

$$\chi^{\alpha\alpha'}_{ij} = \langle c^{\dagger}_{i,\alpha-,\uparrow}c_{j,\alpha'-,\uparrow}\rangle + \langle c^{\dagger}_{i,\alpha-,\downarrow}c_{j,\alpha'-,\downarrow}\rangle. \tag{S15}$$

This leads to the following form of the mean field Hamiltonian, written in matrix form under the basis $\Psi_{\boldsymbol{k}} = (c_{\boldsymbol{k},x+,\uparrow}, c_{\boldsymbol{k},x-,\uparrow}, c_{\boldsymbol{k},z-,\uparrow}, c^{\dagger}_{-\boldsymbol{k},x+,\downarrow}, c^{\dagger}_{-\boldsymbol{k},x-,\downarrow}, c^{\dagger}_{-\boldsymbol{k},z-,\downarrow})^T$:

$$H_{MF} = \sum_{\boldsymbol{k}}\Psi^{\dagger}_{\boldsymbol{k}}H^{\boldsymbol{k}}_{MF}\Psi_{\boldsymbol{k}} = \sum_{\boldsymbol{k}}\Psi^{\dagger}_{\boldsymbol{k}}\begin{pmatrix} \epsilon^{x+}_{\boldsymbol{k}}-\mu & & & & & \\ & \epsilon^{xx}_{\boldsymbol{k}}-\mu & \epsilon^{xz}_{\boldsymbol{k}} & & \Delta^{xx,*}_{\boldsymbol{k}} & \Delta^{xz,*}_{\boldsymbol{k}} \\ & \epsilon^{zx}_{\boldsymbol{k}} & \epsilon^{zz}_{\boldsymbol{k}}-\mu & & \Delta^{zx,*}_{\boldsymbol{k}} & \Delta^{zz,*}_{\boldsymbol{k}} \\ & & & -\epsilon^{x+}_{-\boldsymbol{k}}+\mu & & \\ & \Delta^{xx}_{\boldsymbol{k}} & \Delta^{xz}_{\boldsymbol{k}} & & -\epsilon^{xx}_{-\boldsymbol{k}}+\mu & -\epsilon^{xz}_{-\boldsymbol{k}} \\ & \Delta^{zx}_{\boldsymbol{k}} & \Delta^{zz}_{\boldsymbol{k}} & & -\epsilon^{zx}_{-\boldsymbol{k}} & -\epsilon^{zz}_{-\boldsymbol{k}}+\mu \end{pmatrix}\Psi_{\boldsymbol{k}}. \tag{S16}$$

Here the chemical potential is denoted by $\mu$. The matrix elements are written as follows. For kinetic energy of the bonding orbital:

$$\epsilon^{x+}_{\boldsymbol{k}} = -\sum_{ij} t^{+}_{ij}e^{-i\boldsymbol{k}\cdot\boldsymbol{r}_{ij}}. \tag{S17}$$

Here we use the sum over $ij$ without $\langle ...\rangle$ to denote the site sum over site indices $ij$. The vector connecting $ij$ is denoted as

$\boldsymbol{r}_{ij}=\boldsymbol{r}_i-\boldsymbol{r}_j$. For the two antisymmetric orbitals:

$$\begin{aligned}\epsilon_{\boldsymbol{k}}^{\alpha\alpha'} = &-\sum_{ij} g_t^{\alpha\alpha'} t_{ij}^{\alpha\alpha'} e^{-i\boldsymbol{k}\cdot\boldsymbol{r}_{ij}} - \frac{1}{2U_A}\sum_{\beta}\sum_{ij}\left(t_{ij}^{\beta\alpha'} t_{ij}^{\alpha\alpha'} \chi_{ij}^{\beta\alpha'} - t_{ij}^{\beta\bar{\alpha}'} t_{ij}^{\alpha\bar{\alpha}'} \chi_{ij}^{\beta\alpha'} + 2t_{ij}^{\beta\bar{\alpha}'} t_{ij}^{\alpha\alpha'} \chi_{ij}^{\beta\bar{\alpha}'}\right) e^{-i\boldsymbol{k}\cdot\boldsymbol{r}_{ij}} \\ &- \frac{1}{2U_A}\sum_{\beta}\sum_{ij}\left(t_{ij}^{\beta\alpha} t_{ij}^{\alpha'\alpha} \chi_{ij}^{\beta\alpha} - t_{ij}^{\beta\bar{\alpha}} t_{ij}^{\alpha'\bar{\alpha}} \chi_{ij}^{\beta\alpha} + 2t_{ij}^{\beta\bar{\alpha}} t_{ij}^{\alpha'\alpha} \chi_{ij}^{\beta\bar{\alpha}}\right) e^{i\boldsymbol{k}\cdot\boldsymbol{r}_{ij}}. \end{aligned} \tag{S18}$$

For pairings:

$$\begin{aligned}\Delta_{\boldsymbol{k}}^{\alpha\alpha'} = &-\frac{1}{2U_A}\sum_{\beta}\sum_{ij}\left(2t_{ij}^{\beta\alpha'} t_{ij}^{\alpha\alpha'} \Delta_{ij}^{\beta\alpha'} + t_{ij}^{\beta\alpha'} t_{ij}^{\alpha\bar{\alpha}'} \Delta_{ij}^{\beta\bar{\alpha}'} + t_{ij}^{\beta\bar{\alpha}'} t_{ij}^{\alpha\bar{\alpha}'} \Delta_{ij}^{\beta\alpha'}\right) e^{-i\boldsymbol{k}\cdot\boldsymbol{r}_{ij}} \\ &- \frac{1}{2U_A}\sum_{\beta}\sum_{ij}\left(2t_{ij}^{\beta\alpha} t_{ij}^{\alpha'\alpha} \Delta_{ij}^{\beta\alpha} + t_{ij}^{\beta\alpha} t_{ij}^{\alpha'\bar{\alpha}} \Delta_{ij}^{\beta\bar{\alpha}} + t_{ij}^{\beta\bar{\alpha}} t_{ij}^{\alpha'\bar{\alpha}} \Delta_{ij}^{\beta\alpha}\right) e^{i\boldsymbol{k}\cdot\boldsymbol{r}_{ij}}. \end{aligned} \tag{S19}$$

Here the orbital indices $\alpha\alpha'\beta$ can take either $x$ or $z$. The $\bar{\alpha}$ with the bar refers to the other orbital.

The Hamiltonian can be diagonalized by a unitary transformation $U^{\boldsymbol{k}}$ to get the Bogoliubov-de-Gennes quasiparticle dispersion $E_{n\boldsymbol{k}}$ and the corresponding quasi-particles $d_{n\boldsymbol{k}}$:

$$\Lambda_{\boldsymbol{k}} = U^{\boldsymbol{k}\dagger} H_{MF}^{\boldsymbol{k}} U^{\boldsymbol{k}}, \tag{S20}$$

where $\Lambda_{\boldsymbol{k}}$ is the diagonal matrix of the quasiparticle energy eigenvalue $E_{n\boldsymbol{k}}$. The self-consistent equations can be written in terms of the matrix elements of $U^{\boldsymbol{k}}$, denoted as $u_{in}^{\boldsymbol{k}}$. For pairing mean fields:

$$\begin{aligned}\Delta_{ij}^{xx} &= \langle c_{i,x-,\uparrow}^{\dagger} c_{j,x-,\downarrow}^{\dagger}\rangle - \langle c_{i,x-,\downarrow}^{\dagger} c_{j,x-,\uparrow}^{\dagger}\rangle = \frac{1}{N}\sum_{\boldsymbol{k}}\left[e^{-i\boldsymbol{k}\cdot\boldsymbol{r}_{ij}}\sum_n u_{2n}^{\boldsymbol{k}*} u_{5n}^{\boldsymbol{k}} n_F(E_{n\boldsymbol{k}}) + e^{i\boldsymbol{k}\cdot\boldsymbol{r}_{ij}}\sum_n u_{2n}^{\boldsymbol{k}*} u_{5n}^{\boldsymbol{k}} n_F(E_{n\boldsymbol{k}})\right], \\ \Delta_{ij}^{xz} &= \langle c_{i,x-,\uparrow}^{\dagger} c_{j,z-,\downarrow}^{\dagger}\rangle - \langle c_{i,x-,\downarrow}^{\dagger} c_{j,z-,\uparrow}^{\dagger}\rangle = \frac{1}{N}\sum_{\boldsymbol{k}}\left[e^{-i\boldsymbol{k}\cdot\boldsymbol{r}_{ij}}\sum_n u_{2n}^{\boldsymbol{k}*} u_{6n}^{\boldsymbol{k}} n_F(E_{n\boldsymbol{k}}) + e^{i\boldsymbol{k}\cdot\boldsymbol{r}_{ij}}\sum_n u_{3n}^{\boldsymbol{k}*} u_{5n}^{\boldsymbol{k}} n_F(E_{n\boldsymbol{k}})\right] = \Delta_{ij}^{zx}, \\ \Delta_{ij}^{zz} &= \langle c_{i,z-,\uparrow}^{\dagger} c_{j,z-,\downarrow}^{\dagger}\rangle - \langle c_{i,z-,\downarrow}^{\dagger} c_{j,z-,\uparrow}^{\dagger}\rangle = \frac{1}{N}\sum_{\boldsymbol{k}}\left[e^{-i\boldsymbol{k}\cdot\boldsymbol{r}_{ij}}\sum_n u_{3n}^{\boldsymbol{k}*} u_{6n}^{\boldsymbol{k}} n_F(E_{n\boldsymbol{k}}) + e^{i\boldsymbol{k}\cdot\boldsymbol{r}_{ij}}\sum_n u_{3n}^{\boldsymbol{k}*} u_{6n}^{\boldsymbol{k}} n_F(E_{n\boldsymbol{k}})\right].\end{aligned}$$

Here $n_F(E_{n\boldsymbol{k}})$ denotes the Fermi-Dirac distribution of the quasiparticle with energy $E_{n\boldsymbol{k}}$. The last equality of the second line comes from the ansatz that the pairing is an even function of the orbital index. For hopping mean fields:

$$\begin{aligned}\chi_{ij}^{xx} &= \langle c_{i,x-,\uparrow}^{\dagger} c_{j,x-,\uparrow}\rangle + \langle c_{i,x-,\downarrow}^{\dagger} c_{j,x-,\downarrow}\rangle = \frac{1}{N}\sum_{\boldsymbol{k}}\left[e^{-i\boldsymbol{k}\cdot\boldsymbol{r}_{ij}}\sum_n u_{2n}^{\boldsymbol{k}*} u_{2n}^{\boldsymbol{k}} n_F(E_{n\boldsymbol{k}}) + e^{i\boldsymbol{k}\cdot\boldsymbol{r}_{ij}}\sum_n u_{5n}^{\boldsymbol{k}} u_{5n}^{\boldsymbol{k}*}(1-n_F(E_{n\boldsymbol{k}}))\right], \\ \chi_{ij}^{xz} &= \langle c_{i,x-,\uparrow}^{\dagger} c_{j,z-,\uparrow}\rangle + \langle c_{i,x-,\downarrow}^{\dagger} c_{j,z-,\downarrow}\rangle = \frac{1}{N}\sum_{\boldsymbol{k}}\left[e^{-i\boldsymbol{k}\cdot\boldsymbol{r}_{ij}}\sum_n u_{2n}^{\boldsymbol{k}*} u_{3n}^{\boldsymbol{k}} n_F(E_{n\boldsymbol{k}}) + e^{i\boldsymbol{k}\cdot\boldsymbol{r}_{ij}}\sum_n u_{5n}^{\boldsymbol{k}} u_{6n}^{\boldsymbol{k}*}(1-n_F(E_{n\boldsymbol{k}}))\right], \\ \chi_{ij}^{zz} &= \langle c_{i,z-,\uparrow}^{\dagger} c_{j,z-,\uparrow}\rangle + \langle c_{i,z-,\downarrow}^{\dagger} c_{j,z-,\downarrow}\rangle = \frac{1}{N}\sum_{\boldsymbol{k}}\left[e^{-i\boldsymbol{k}\cdot\boldsymbol{r}_{ij}}\sum_n u_{3n}^{\boldsymbol{k}*} u_{3n}^{\boldsymbol{k}} n_F(E_{n\boldsymbol{k}}) + e^{i\boldsymbol{k}\cdot\boldsymbol{r}_{ij}}\sum_n u_{6n}^{\boldsymbol{k}} u_{6n}^{\boldsymbol{k}*}(1-n_F(E_{n\boldsymbol{k}}))\right].\end{aligned}$$

And the particle number can be calculated according to:

$$\begin{aligned}n_{x+} &= \langle c_{i,x+,\uparrow}^{\dagger} c_{i,x+,\uparrow}\rangle + \langle c_{i,x+,\downarrow}^{\dagger} c_{i,x+,\downarrow}\rangle = \frac{1}{N}\sum_{n\boldsymbol{k}}\left[u_{1n}^{\boldsymbol{k}*} u_{1n}^{\boldsymbol{k}} n_F(E_{n\boldsymbol{k}}) + u_{4n}^{\boldsymbol{k}} u_{4n}^{\boldsymbol{k}*}(1-n_F(E_{n\boldsymbol{k}}))\right], \\ n_{x-} &= \langle c_{i,x-,\uparrow}^{\dagger} c_{i,x-,\uparrow}\rangle + \langle c_{i,x-,\downarrow}^{\dagger} c_{i,x-,\downarrow}\rangle = \frac{1}{N}\sum_{n\boldsymbol{k}}\left[u_{2n}^{\boldsymbol{k}*} u_{2n}^{\boldsymbol{k}} n_F(E_{n\boldsymbol{k}}) + u_{5n}^{\boldsymbol{k}} u_{5n}^{\boldsymbol{k}*}(1-n_F(E_{n\boldsymbol{k}}))\right], \\ n_{z-} &= \langle c_{i,z-,\uparrow}^{\dagger} c_{i,z-,\uparrow}\rangle + \langle c_{i,z-,\downarrow}^{\dagger} c_{i,z-,\downarrow}\rangle = \frac{1}{N}\sum_{n\boldsymbol{k}}\left[u_{3n}^{\boldsymbol{k}*} u_{3n}^{\boldsymbol{k}} n_F(E_{n\boldsymbol{k}}) + u_{6n}^{\boldsymbol{k}} u_{6n}^{\boldsymbol{k}*}(1-n_F(E_{n\boldsymbol{k}}))\right].\end{aligned}$$

In calculations, the parameters are chosen as follows. The Hubbard interaction $U_A = 6$ and the in-plane nearest neighbor hoppings are given by $t^{xx} = 1$, $t^{zz} = 0.2$ according to [27] for the case of $La_3Ni_2O_7$ . The interorbital hoppings $t^{xz} = 0.1$

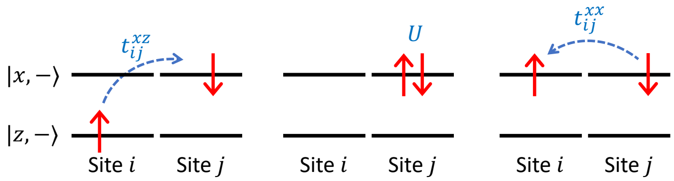


FIG. S1. Virtual hopping process corresponding to the interaction term $c_{ix\uparrow}^{\dagger}c_{jx\downarrow}^{\dagger}c_{jx\downarrow}c_{iz\uparrow}$, leading to the hybrid pairing interaction of the form $\Delta^{xx,\dagger}\Delta^{xz}$ with interaction strength $\propto t_{ij}^{xz}t_{ij}^{xx}/U_A$.

and $t^{xz} = 0.8$ are considered. Moreover, the calculations are performed with varying number of self-doped holes $n_h$, which corresponds to varying the molecular energy splitting $\epsilon_{x+}$. The total electron number is fixed by the chemical potential $\mu$ to yield $\langle n\rangle = \langle n_h\rangle + \langle n_{x-}\rangle + \langle n_{z-}\rangle = 1$ without external holes.

**pairing interaction from the two-orbital process**

The pairing part of the Hamiltonian takes the form:

$$H_{\text{pairing}} = \sum_{ij}\sum_{\alpha\alpha'\beta}\left[-\frac{t_{ij}^{\alpha\beta}t_{ij}^{\alpha'\beta}}{2U_A}\left(2\Delta_{ij}^{\alpha'\beta,\dagger}\Delta_{ij}^{\alpha\beta} + \Delta_{ij}^{\alpha'\bar{\beta},\dagger}\Delta_{ij}^{\alpha\bar{\beta}}\right) - \frac{t_{ij}^{\alpha\beta}t_{ij}^{\alpha'\bar{\beta}}}{2U_A}\Delta_{ij}^{\alpha'\beta,\dagger}\Delta_{ij}^{\alpha\bar{\beta}}\right]. \tag{S21}$$

By further considering the orbital indices, the pairing part can be divided into the intra-orbital, inter-orbital as well as hybrid channels (sum over $\alpha$ is implied):

$$H_{\text{pairing}}^{\text{intra-orbital}} = -\frac{t_{ij}^{\alpha\alpha}t_{ij}^{\alpha\alpha}}{U_A}\Delta_{ij}^{\alpha\alpha,\dagger}\Delta_{ij}^{\alpha\alpha} - \frac{t_{ij}^{\alpha\bar{\alpha}}t_{ij}^{\alpha\bar{\alpha}}}{2U_A}\Delta_{ij}^{\alpha\alpha,\dagger}\Delta_{ij}^{\alpha\alpha} - \frac{t_{ij}^{\bar{\alpha}\alpha}t_{ij}^{\alpha\bar{\alpha}}}{2U_A}\Delta_{ij}^{\alpha\alpha,\dagger}\Delta_{ij}^{\bar{\alpha}\bar{\alpha}}, \tag{S22}$$

$$H_{\text{pairing}}^{\text{inter-orbital}} = -\frac{t_{ij}^{\alpha\bar{\alpha}}t_{ij}^{\alpha\bar{\alpha}}}{U_A}\Delta_{ij}^{\alpha\bar{\alpha},\dagger}\Delta_{ij}^{\alpha\bar{\alpha}} - \frac{t_{ij}^{\alpha\alpha}t_{ij}^{\alpha\alpha}}{2U_A}\Delta_{ij}^{\alpha\bar{\alpha},\dagger}\Delta_{ij}^{\alpha\bar{\alpha}} - \frac{t_{ij}^{\alpha\alpha}t_{ij}^{\bar{\alpha}\bar{\alpha}}}{2U_A}\Delta_{ij}^{\bar{\alpha}\alpha,\dagger}\Delta_{ij}^{\alpha\bar{\alpha}}, \tag{S23}$$

$$H_{\text{pairing}}^{\text{hybrid}} = -\frac{t_{ij}^{\alpha\alpha}t_{ij}^{\bar{\alpha}\alpha}}{U_A}\Delta_{ij}^{\bar{\alpha}\alpha,\dagger}\Delta_{ij}^{\alpha\alpha} - \frac{t_{ij}^{\alpha\alpha}t_{ij}^{\bar{\alpha}\alpha}}{2U_A}\Delta_{ij}^{\bar{\alpha}\bar{\alpha},\dagger}\Delta_{ij}^{\alpha\bar{\alpha}} - \frac{t_{ij}^{\bar{\alpha}\alpha}t_{ij}^{\alpha\alpha}}{2U_A}\Delta_{ij}^{\alpha\bar{\alpha},\dagger}\Delta_{ij}^{\alpha\alpha} + \text{h.c.}. \tag{S24}$$

The last equation is unique to the considered two orbital interactions. Taking the first term in the last line, $-\frac{t_{ij}^{\alpha\alpha}t_{ij}^{\bar{\alpha}\alpha}}{U_A}\Delta_{ij}^{\bar{\alpha}\alpha,\dagger}\Delta_{ij}^{\alpha\alpha}$, and set $\alpha = x$, $\bar{\alpha} = z$ as an example, the virtual process giving this term can be depicted in Fig. S1.
